# Motor Imagery Classification Based on EEG Sensing with Visual and Vibrotactile Guidance

**DOI:** 10.3390/s23115064

**Published:** 2023-05-25

**Authors:** Luka Batistić, Diego Sušanj, Domagoj Pinčić, Sandi Ljubic

**Affiliations:** 1 University of Rijeka, Faculty of Engineering, Vukovarska 58, HR-51000 Rijeka, Croatia; lbatistic@riteh.hr (L.B.); dpincic@riteh.hr (D.P.); 2 Center for Artificial Intelligence and Cybersecurity, University of Rijeka, R. Matejcic 2, HR-51000 Rijeka, Croatia

**Keywords:** BCI, EEG, motor imagery, somatosensory guidance, machine learning

## Abstract

Motor imagery (MI) is a technique of imagining the performance of a motor task without actually using the muscles. When employed in a brain–computer interface (BCI) supported by electroencephalographic (EEG) sensors, it can be used as a successful method of human–computer interaction. In this paper, the performance of six different classifiers, namely linear discriminant analysis (LDA), support vector machine (SVM), random forest (RF), and three classifiers from the family of convolutional neural networks (CNN), is evaluated using EEG MI datasets. The study investigates the effectiveness of these classifiers on MI, guided by a static visual cue, dynamic visual guidance, and a combination of dynamic visual and vibrotactile (somatosensory) guidance. The effect of filtering passband during data preprocessing was also investigated. The results show that the ResNet-based CNN significantly outperforms the competing classifiers on both vibrotactile and visually guided data when detecting different directions of MI. Preprocessing the data using low-frequency signal features proves to be a better solution to achieve higher classification accuracy. It has also been shown that vibrotactile guidance has a significant impact on classification accuracy, with the associated improvement particularly evident for architecturally simpler classifiers. These findings have important implications for the development of EEG-based BCIs, as they provide valuable insight into the suitability of different classifiers for different contexts of use.

## 1. Introduction

Medical conditions and injuries that limit the ability to perform motor activities can have a profound impact on the lives of those affected. As a result, numerous research groups are attempting to restore or replace lost abilities through various means. A brain–computer interface (BCI) that uses electroencephalography (EEG) is one such technique. EEG is an electrophysiological monitoring technique that records the electrical activity of the brain by detecting the potentials associated with cortical neural activity. Other electrophysiological monitoring methods used to record the activity of our brain include electrocorticography (ECoG), functional magnetic resonance imaging (fMRI), electromiogram (EMG), magnetoencephalography (MEG), and near-infrared spectroscopy (NIRS). EEG is a noninvasive method, which means that electrodes are placed along the scalp. ECoG is a similar but invasive method, usually with electrodes placed directly on the exposed surface of the brain. Local field potential (LFP) is a signal recorded from adjacent neurons within a small volume of nervous tissue. fMRI is a neuroimaging method that assesses brain activity by detecting changes in blood flow associated with neural activation. EMG records the electrical activity of skeletal muscles in order to evaluate their function, whereas MEG records the magnetic fields generated by neural activity, allowing for a high-resolution examination of brain dynamics. NIRS is an optical imaging technique that utilizes low levels of light to measure changes in blood oxygenation levels in the brain, thereby providing information regarding neural activity. EEG can be used to operate or communicate to a computer without the use of neuromuscular pathways and is practical due to its portability and affordability. A BCI detects the user’s intention by preprocessing the electrophysiologically recorded data [1]. BCI research to date has focused primarily on the communication element using event-related potentials (e.g., P300 responses) [2,3,4,5], steady-state visual evoked potentials (SSVEP) [6,7,8,9], and sensorimotor rhythms [10,11,12]. Nevertheless, BCIs have significant development potential for controlling physical devices [13,14,15,16,17,18,19].

The rhythmic activity of the brain recorded in EEG, ECoG, LFP, and MEG via the sensorimotor cortex is influenced by movement, intention to move, or imagining movement, which is an important observation. The influence manifests as a decrease in power in the alpha or mu (8–13 Hz) and beta (14–26 Hz) frequency bands, also called event-related desynchronization (ERD), which is accompanied by an increase in power in the gamma frequency band (>30 Hz), also called event-related synchronization (ERS) [20]. These rhythmic activities are referred to as sensorimotor rhythms (SMR) [21]. Kobler et al. [22] demonstrated that direction information is also contained in the low-frequency delta band (0.2–5 Hz). Motor intention or motor imagery can be captured by SMR-based BCI, which is the basis for brain control in such systems. Several studies have shown that individuals can learn to control the amplitude of SMR via MI [10,23,24,25]. In several experiments, subjects were able to achieve both 2D and 3D control [10,25]. The sources of sensorimotor rhythms triggered by movements or imagined movements of different body parts were localized in the primary sensorimotor cortex in a somatotopic manner [26].

EEG data are complex, nonstationary, multicomponent signals that represent the brain’s dynamic electrical activity [27,28]. The nonstationarity of EEG signals means that their statistical properties, such as mean and variance, can vary over time. This inherent complexity and temporal variability can complicate the analysis and interpretation of EEG signals. Moreover, as multicomponent signals, EEG data reflect the superimposed activity of multiple neural sources that can vary independently and contribute to the nonstationarity of the signals as a whole. According to recent research by Miladinović et al. [29], the nonstationarity of EEG signals can lead to shifts in feature covariance over time, requiring the use of specialized analytical techniques to accurately capture and interpret the changing dynamics of these signals.

Many EEG targets (such as stimulus-evoked potentials and slow cortical potentials) are recorded noninvasively, including rhythmic activity over the sensorimotor cortex. SMR has been shown to be a stable and valid EEG target for improving motor skills in healthy individuals as well as in individuals with motor impairments using various BCI and neurofeedback (NF) techniques [30]. Although BCI performance varies for different users, the majority of users (up to 80%) are able to operate BCI with sufficient accuracy by voluntarily modulating SMR amplitude (e.g., by imagining movements) [31]. In addition, patients with motor difficulties (even with amyotrophic lateral sclerosis [32]) have been shown to potentially achieve SMR modulations that serve as a platform for BCI systems that extract patient intentions from EEGs and convert them into control signals for prostheses or computer applications [31,33]. Task-related SMR modulation is often detected as an amplitude decrease in the low-frequency range (alpha/beta/mu bands). Both planning (imagining) and execution of limb movements have been shown to result in predictable decreases in the alpha/beta frequency bands, organized in a somatotopic manner [20,21].

### 1.1. Related Work

Some research efforts have focused on proprioceptive kinesthetic feedback of movement execution (ME) and the lack of proprioceptive kinesthetic feedback in MI [34,35,36,37]. In natural movement processes, movement execution and feedback processes (such as haptic information, proprioception, and visual information) cannot be treated as independent. Rather, movement-related actions are changed and corrected throughout execution based on sensory input, which enhances BCI control performance [37,38]. In addition to visual guidance, vibrotactile guidance is used as sensory input in one of the datasets analyzed in this research.

Although many different methods are used for classification in MI data (which are nonstationary multicomponent signals [29]), linear discriminant analysis (LDA) and support vector machine (SVM) are commonly utilized. The random forest (RF) classifier has been investigated and proven to give similar results to the SVM and LDA. Convolutional neural networks (CNNs) are a rapidly developing machine learning method that can be used as a classification tool and have recently been explored with growing interest in the field of BCI. These methods are applied and tested on the MI datasets, with results presented in this paper.

Contemporary research efforts are focused on the use of machine learning techniques for EEG MI. Many studies have demonstrated the effectiveness of LDA in various applications, including EEG-based BCIs for MI classification [36,37]. However, LDA and its variants are often used as a benchmark for BCI research from an experimental and neurophysiological perspective (new experimental paradigms, new patterns, or new types of signals acquired in BCIs) [37,38,39,40]. Ofner et al. [39] reported accuracy around the chance level, but for six-class problems, and Hehenberger et al. [38] reported an average accuracy of about 64% for the sLDA classifier when classifying different MIs of the same limb.

In the case when SVM was utilized, Vargic et al. [41] achieved accuracy between 47.86% and 70.71% in classifying MI of different limbs. Ma et al. [42] reported even better results, achieving accuracy between 76% and 91%, also for MI of different limbs.

When it comes to classifying MI of different limbs using the RF method, Zhang et al. [43] reported accuracies of up to 76%, Steyrl et al. [40] have reported accuracies of up to 79.30% when utilizing common spatial patterns and RF, and Bentlemsan et al. [44] have achieved an accuracy of 79.77%.

Finally, research based on the use of neural networks is also predominantly concerned with the MI classification of different limbs. For example, Zhang et al. [45] have shown an accuracy of 90% in classifying MI of the right and left hand by using a wavelet neural network and artificially augmented data. Hou et al. [46] achieved an accuracy of 93.06% by using graph convolutional neural networks (GCNs) in classifying MI of the right hand, left hand, fists, and feet. Strahnen and Kessler [47] used a deep neural network (DNN) and achieved up to 80.7% accuracy in classifying MI of cyclic opening/closing of the left or right fist. On the other hand, Lee et al. [48] used a channel-wise variational autoencoder CNN to classify data from Ofner et al. [39] and achieved up to 60% accuracy in classifying the *elbow extension* class against other different MIs of the same limb.

### 1.2. Contributions and Structure

Related work shows that higher classification accuracy is easier to achieve for different-limb MI than for same-limb MI. However, classifying the same-limb MI is more challenging than classifying different-limb MI, because performing different motor tasks with one limb usually activates the same brain regions [49,50]. Despite the increased complexity in classification, same-limb MI offers potential benefits, such as more precise and intuitive control in neuroprosthetic applications [49,51], making it a valuable area of research. In addition, improving classification accuracy in same-limb MI could greatly enhance the performance and reliability of EEG-based BCIs and open new possibilities for their application in areas such as neurorehabilitation and assistive technology, especially when patients have suffered unilateral lesions and cannot function equally well with both halves of the body [52]. The end-user device for a project involving such same-limb MI classification with kinesthetic feedback could be a robotic arm with intuitive control, the practical implications of which are described conceptually by Müller-Putz et al. [53].

For same-limb MI classification, solutions based on simpler ML methods are often used and achieve accuracy ranging from a questionably useful chance-level (about 50% for the two-class problem) to as high as 64%. To the best of our knowledge, there is no research that applies contemporary ML techniques with more complex architectures for classifying same-limb different MIs. In our study, we selected MI tasks that refer to different movements of the same limb, because these tasks are often required in real-world BCI use cases, have distinct feature differences, and pose a major challenge for motion classification. Thus, we can directly address the identified research gap in our study. We provide the following contributions to the field of ML-supported EEG analysis:

- Introduction of a new application for specific deep CNNs to classify the same-limb MI, with the aim of improving classification accuracy beyond the state-of-the art;
- Benchmarking and in-depth performance analysis for various classification methods, including the commonly used and the more architecturally complex newly applied methods;
- In-depth statistical analyses of the effects of different guidance techniques (visual guidance or a combination of visual and vibrotactile guidance) and different data preprocessing on classification accuracy for all observed methods.

The rest of the paper is organized as follows. Section 2 presents the datasets used and the details of the experiments that led to their creation, as well as the data preprocessing procedure and a formal description of all classification methods used in our research. Section 3 provides a detailed presentation of the results, a related discussion, and general conclusions based on the statistical tests performed. Concluding remarks are presented in Section 4.

## 2. Materials and Methods

In this section, we describe the datasets used in our study as well as the preprocessing steps that were applied to the data. We then present the details of the classification methods that were employed.

### 2.1. Datasets

In this study, we developed and evaluated our approaches using two datasets. The first dataset is the MI dataset obtained from the BNCI Horizon 2020 project [39]. For simplicity and clarity, we refer to this dataset as ULM in this paper. This reflects the fact that it originates from research in which Upper Limb Movements were used. The ULM was chosen to test our approaches on one of the most widely used MI datasets available online. In addition, this dataset was chosen for its simplicity (we selected two simple linear continuous center-out MIs of the same limb: *elbow flexion* and *elbow extension*).

The second dataset was curated as part of the “Feel Your Reach” project funded by the European Research Council and carried out by the Institute of Neural Engineering at the Graz University of Technology [38,53]. For simplicity and clarity, we refer to this dataset as KGU in this paper. This reflects the fact that it comes from research wherein Kinesthetic GUidance was introduced into the paradigm. We chose this very dataset for its simplicity (linear continuous center-out MI, direction *up* and direction *right*) and diversity (two conditions: MI with vibrotactile guidance and MI without vibrotactile guidance). Although the experiment of the KGU dataset employs visual guidance and the experiment of the ULM dataset employs visual cues, and the experiment of the KGU dataset employs two different conditions, its simple MI tasks and paradigm are quite comparable to those of the ULM dataset.

Although the ULM and KGU datasets were not originally curated as part of this study, in Section 2.1.1 and Section 2.1.2, we will describe the details of the data collection procedure and the experiment paradigm for both datasets, as these details are essential to our research.

#### 2.1.1. ULM Dataset

**Figure 1:**
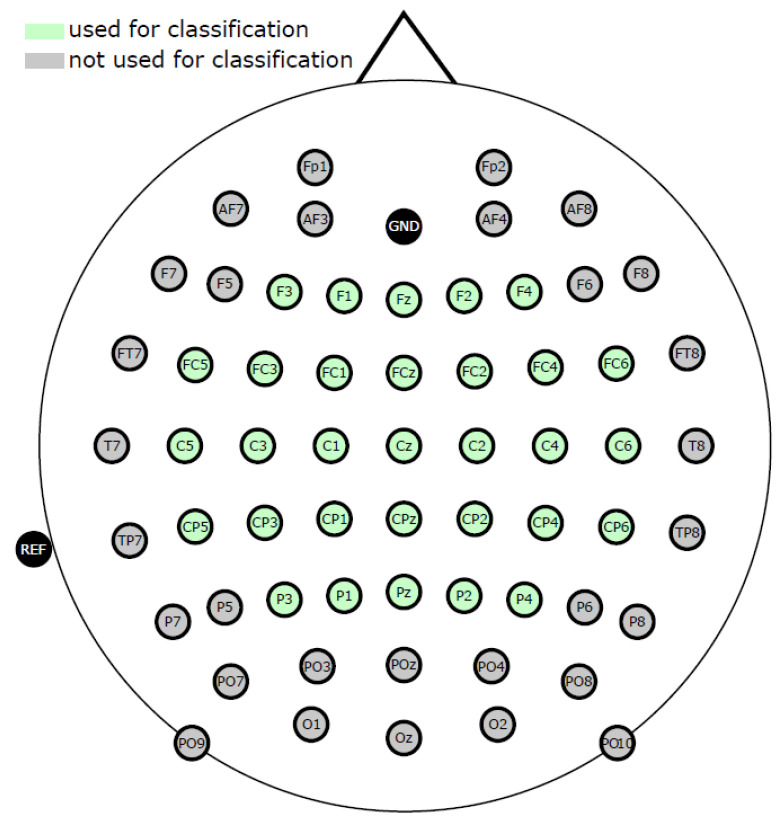
International 10/20 EEG system cap montage [38].

Four g.tec amplifiers (g.tec medical engineering GmbH, Austria) with a sampling rate of 512 Hz were used to capture EEG and EOG from 61 and 3 active electrodes, respectively. Between 0.01 Hz and 200 Hz, an eighth-order Chebyshev bandpass filter was applied to the dataset. Only the 31 electrodes highlighted in green (see Figure 1) conformed to the international 10/20 EEG system cap montage. Because of these disparities in montage, we used only the corresponding EEG and EOG electrodes for preprocessing, analysis, and classification for the ULM dataset.

**Figure 2:**
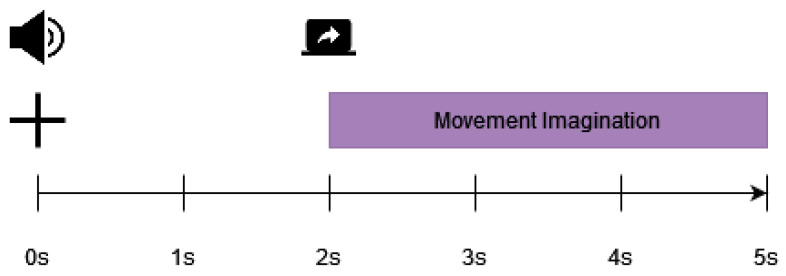
Paradigm of the experiment trial of ULM dataset [39].

Data were curated from 15 participants, 6 men and 9 women, aged between 22 and 40 (mean 27, standard deviation = 5). All but one individual were right-handed. Each participant completed an MI session in which ten runs were recorded. Each run consisted of different MI tasks (elbow flexion, elbow extension, supination, pronation, hand closing, hand opening) in which static visual cues were displayed on the screen in front of the participant. Each run consisted of 36 trials (6 of each 6 tasks). As illustrated in Figure 2, each trial lasted 5 s, and MI occurred within a 3 s interval. The fixation cross was displayed for 2 s, with the last 1.5 s being used as the baseline period (for preprocessing). Participants were told to fixate their eyes on the fixation cross during this interval. The monitor then provided a stationary visual cue to the required action (one of six movements). Participants were instructed to perform the MI based on the stationary visual cue [39]. Only two of the above tasks were used in our research: *elbow flexion* (EF) and *elbow extension* (EE). These tasks were selected based on their similarity to those in the KGU dataset (MI *up* and *right*).

#### 2.1.2. KGU Dataset

EEG and EOG were recorded at a sampling rate of 1 kHz from 61 electrodes and 3 actiCap electrodes using two BrainAmp amplifiers (Brain Products GmbH, Gilching, Germany). Electrodes were set according to the international 10/20 EEG standard, as shown in Figure 1, with 61 channels for EEG and three channels for EOG. In the subsequent preprocessing and analysis of the data, only 31 channels surrounding motor-related regions (indicated in green in Figure 1) were used.

Data were curated from 15 participants. Participants were between 21 and 32 years old (mean 25.36, standard deviation 3.4); 7 were men and 8 were women. All participants were right-handed. Ten of the fifteen individuals had previous experience with MI. Each participant took part in a single session in which six runs were recorded. Each run included either VtG (visual guidance and vibrotactile guidance) or noVtG (visual guidance only) MI tasks, totaling three VtG and three noVtG runs. In the VtG condition, vibrotactile guidance was provided by three tactile actuators (C-2 tactors—Engineering Acoustics Inc., Casselberry, FL, USA) attached to the inside of an elastic shirt to stimulate the right shoulder blade [38]. These tactors provided vibrotactile haptic guidance through a moving sensation on the participant’s shoulder. Each run consisted of forty trials.

**Figure 3:**
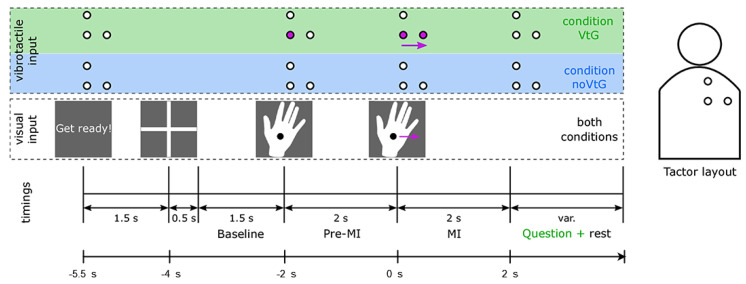
Paradigm of the experiment trial of the KGU dataset [38]. The top row of the image (shaded in green and blue) depicts the position and activation of the tactors that delivered vibrotactile input (guidance) in congruence with the visual input (depicted in the middle row of the image). Timings are shown in the bottom row of the image.

As can be seen in Figure 3, each trial lasted 7.5 s and MI occurred over a 2 s interval. At the beginning of each trial, 1.5 s before the fixation cross was displayed, participants were visually instructed to “Get ready”. The fixation cross was presented for 2 s, with the last 1.5 s used as the baseline period (later utilized for preprocessing and classification). Participants were asked to focus their eyes on the fixation cross and to relax during this period. Then, the visual cue was displayed on the monitor—a right hand with a fixation point. During the two seconds preceding MI, it stayed fixed before starting to move either to the right or upward at a constant pace. Participants were told to perform MI in accordance with the cue’s movement and to focus their eyes on a fixation point (a black dot in the middle of the hand cue). In the VtG condition, participants were also asked to decide whether the vibrotactile guidance in this trial was consistent with the visual guidance by pressing the (keyboard) key [38] in response.

In this experiment, the goal of adding vibrotactile stimulation is to replicate the proprioceptive kinesthetic feedback that the subject would feel in the event of movement execution. In the VtG condition, the subject performed the MI and at the same time vibrotactile stimulation was present (giving the same directional information as the imagined movement). Hence, for each class in the KGU dataset, there are two different feature types: MI with vibrotactile stimulation (VtG) and MI without vibrotactile stimulation (noVtG).

The KGU dataset differs from the ULM dataset in terms of the timing of the paradigms, imagined movements (*elbow flexion* and *elbow extension* for the ULM dataset, *up* and *right* for the KGU dataset), and the presence of vibrotactile guidance in certain trials of the KGU dataset’s experiment paradigm. While the experiment of the KGU dataset involves visual guidance, the experiment of the ULM dataset involves only a stationary visual cue.

### 2.2. Data Preprocessing

Before proceeding to classification, the data were preprocessed as follows:

1. The data were downsampled to 200 Hz, and a fourth-order zero-phase Butterworth bandpass filter was utilized with a passband between 1 and 40 Hz, epoched to the relevant time period (from t=0 s to t=5 s, as shown in Figure 2 for the ULM dataset, and from t=−5.5 s to t=2 s, as shown in Figure 3 for the KGU dataset).
2. Bad trials, based on amplitude threshold and artifact presence, were rejected using the EEGLAB Matlab toolbox [54].
3. Independent component analysis (ICA) [54,55] was performed separately for each participant. For the ULM dataset, it was performed for 31 EEG channels (yielding 31 independent components). The remaining 3 EOG channels were used for artifact removal. For the KGU dataset, it was performed for 61 EEG channels (yielding 61 independent components). In this case, the EOG channels were also used for artifact removal. For both datasets, only relevant independent components (IC) were retained, using SASICA [56] and manual IC rejection.
4. The data were further filtered (fourth-order zero-phase Butterworth filter) in the bands of interest, specifically 0.2–5 Hz for low-frequency features and 1–40 Hz for broad-frequency features. Only relevant MI periods were epoched for classification (from t=2 s to t=4 s for the ULM dataset, as shown in Figure 2, and from t=0 s to t=2 s for the KGU dataset, as shown in Figure 3).
5. The features were then further downsampled to 20 Hz for low-frequency features and to 100 Hz for broad-frequency features.
6. In total, 31 relevant channels around the motor-related area were selected for further preprocessing and analysis (Figure 1).

Throughout the classification phase of our study, these features were utilized to apply and evaluate the various classification methods.

### 2.3. Classification

For all methods tested, classification was performed using the same features for each subject separately. The features were obtained from the preprocessing phase (Section 2.2). Each of the two classes had 60 trials for the ULM dataset or 120 trials for the KGU dataset. Each trial is represented by the *T* matrix, whose dimension is NC×NS, where NC=31 is the number of channels and NS is the number of samples during the MI period.

For sLDA, SVM, and RF, a sliding window with a size of WS corresponding to the time period of 0.5 s was used to calculate the feature matrix $F_{i}$ throughout the trial. The resulting $F_{i}$ matrix has dimension NC×WS. The feature vector $\vec{f_{i}}$ with NC features represents row-wise averages from the matrix $F_{i}$ and is used for classification. Using the classification accuracy results from all subjects, average classification accuracy is calculated for each time point *i* in the trial. From this, a time point with a peak (maximum) of the average classification accuracy is calculated. The resulting classification accuracy for each subject is determined as the accuracy value at the said peak time point.

During our preliminary investigations, we found that the CNN-based methods with the window-based approach had much more consistent accuracy within the intended time frame. Therefore, we decided to use the entire trial matrix *T* for classification with CNN models. Because of this consistency of the CNN classifiers, there is no difference in the accuracy achieved between the approach using the entire trial matrix and the window-based approach. In summary, the feature vector $\vec{f_{i}}$ with 31 components representing the mean of each EEG channel for each of the 0.5 s windows is used as input for sLDA, SVM, and RF. For CNN-based methods, the entire trial matrix *T* was used.

For all methods, five-fold cross-validation was performed, with 80% of the dataset used for training and 20% used for testing in each cross-validation. The classification methods are compared based on the average accuracy they achieved. As for the implementation of these methods in our study, we used standard Matlab functions for sLDA, SVM, and RF, while PyTorch was used for CNN-based methods.

#### 2.3.1. Shrinkage Linear Discriminant Analysis

Linear Discriminant Analysis (LDA) is a classification algorithm used to model the differences between groups or classes based on their input features. LDA first identifies the linear feature combinations that best distinguish groups from one another. These linear combinations are then used to project the data onto a lower-dimensional space where groups can be more easily separated. In this lower-dimensional space, LDA constructs a linear boundary, or decision boundary, that separates the classes. To classify new observations, LDA projects the observation onto this decision boundary and assigns it to the class on the corresponding side. LDA assumes that the input features are normally distributed and that the covariance matrix is equal across groups [57]. LDA is widely used in various applications, including image recognition, text classification, and bioinformatics.

Shrinkage Linear Discriminant Analysis (sLDA) (see Blankertz et al. [58]) is an extension of LDA designed to improve its performance in high-dimensional environments. For high-dimensional data, traditional LDA may suffer from overfitting, i.e., it may not generalize well to new data. The main idea behind shrinkage LDA is to estimate a “shrinkage” covariance matrix, which is a compromise between the sample covariance matrix and a diagonal matrix. This shrinkage matrix is computed as a linear combination of the sample covariance matrix and a diagonal matrix, with the weighting of the sample covariance matrix determined by the shrinkage parameter. By shrinking towards a diagonal matrix, the algorithm reduces the number of parameters to be estimated, which in turn reduces the risk of overfitting.

The classification procedure in sLDA can be summarized as follows:

1. Calculate the shrinkage LDA coefficients, i.e., the weights that define the linear boundary between the different classes.
2. Calculate the class means for each group.
3. Calculate the pooled within-class covariance matrix, which is a weighted sum of the sample covariance matrices for each group.
4. Calculate the shrinkage covariance matrix $\Sigma_{s}$ using the formula:
  (1) $$\Sigma_{s}=\left(1-\delta \right)S+\delta D,$$
  where *S* is the sample covariance matrix, *D* is the diagonal matrix, and δ is the shrinkage parameter.
5. Calculate the inverse of the pooled within-class covariance matrix.
6. Calculate the discriminant value D(x) for each observation using the formula:
  (2) $$D\left(x\right)=x^{T}\Sigma_{w}^{-1}\left(\mu_{1}-\mu_{2}\right),$$
  where *x* is the feature vector, $\Sigma_{w}^{-1}$ is the inverse of the pooled within-class covariance matrix, and $\mu_{1}$ and $\mu_{2}$ are the means of the two classes.
7. Classify each observation based on the sign of the discriminant value. If D(x)>0, the observation is classified as belonging to the first class. If D(x)≤0, then the observation is classified as belonging to the second class.

#### 2.3.2. Support Vector Machine

Support Vector Machines (SVM) is a classification algorithm that finds the best hyperplane in a high-dimensional space that separates the classes with the largest margin. The margin is the distance between the hyperplane and the closest data points of each class, and SVM tries to find the hyperplane that maximizes this margin [59,60]. If the data are not linearly separable, SVM uses a kernel function to map the data into a higher-dimensional space where it is more likely to be linearly separable. In this higher-dimensional space, SVM constructs a hyperplane that separates the classes with the largest margin. SVM can be used for both binary and multi-class classification tasks.

Kernel SVM is a variant of the SVM in which a kernel function is used to map the data into a higher-dimensional space, where it can be more easily separated by a hyperplane. The kernel function computes the dot product between two feature vectors in this higher-dimensional space without actually computing the coordinates of the vectors in that space. This is known as the “kernel trick” and allows the SVM to work efficiently with high-dimensional data. The kernel SVM is particularly useful when the data are not linearly separable. Typically, various kernel functions are used, such as the linear function, polynomial function, radial basis function (RBF), and sigmoid function.

The classification procedure in kernel SVM can be summarized as follows:

1. Given a set of training data, the kernel SVM selects a subset of data points as support vectors, i.e., the points closest to the decision boundary in higher-dimensional space.
2. The kernel SVM then finds the hyperplane that maximizes the distance between the support vectors of each class in the higher-dimensional space.
3. To classify new observations, the kernel SVM maps them into higher-dimensional space using the kernel function, projects them onto the hyperplane, and assigns them to the class on the corresponding side. The sign of the projection determines the class of the observation.

#### 2.3.3. Random Forest

The RF method uses a group of predictors (e.g., decision trees) in order to aggregate the prediction votes and then predict the outcome that gets the most votes. The group of predictors is called an ensemble; thus, methods such as RF are called ensemble methods. In our case, we use RF with 20 decision trees (DTs) [60,61]. In this method, multiple decision trees are built using a random subset of the training data and a random subset of the input features. The final prediction is made by averaging the predictions of all the trees in the forest. RF is known for its ability to handle high-dimensional data and its robustness to noise, making it a suitable choice for EEG data. The method works as follows:

1. Randomly sample the training data with replacement (bootstrap) to create multiple datasets (or decision trees) of the same size as the original dataset.
2. For each dataset, randomly select a subset of the input features to use for building the DT.
3. Build a DT for each dataset using the selected features and a splitting criterion.
4. Repeat steps 1–3 to create a forest of DT.
5. To make a prediction for a new sample, pass it through all the DTs in the forest and average their predictions (for regression tasks) or take the majority vote (for classification tasks).

RF has several advantages over other machine learning methods. It can handle high-dimensional data with many input features and is less prone to overfitting than DTs. The method is also able to capture nonlinear relationships and interactions between input features. In addition, RF is computationally efficient and can be easily parallelized, making it suitable for large datasets.

One way to obtain a diverse set of classifiers in ensemble methods (e.g., RF) is to use very different training methods. Another approach is to use the same training method for each predictor, but train them on different random subsets of the training set. When sampling is performed with replacement, this method is called bagging. Bagging allows training instances to be sampled several times across multiple predictors. Our final implementation of RF uses bagging and 20 DTs.

#### 2.3.4. VGG-19

The VGG-19 architecture [62] is a deep CNN known for its simplicity, depth, and strong performance in various computer vision tasks, such as image classification and object detection.

VGG-19 consists of 19 layers: 16 convolutional layers and 3 fully connected layers. These layers are organized into five convolutional blocks and one fully connected block. Each convolutional block contains a varying number of convolutional layers with small 3×3 filters, followed by a max-pooling layer. The fully connected block consists of three fully connected layers, followed by a softmax activation function [63] to output class probabilities.

Convolution allows the model to learn local patterns and spatial features in the data by sliding a filter *K* over the input data, computing an element-wise multiplication between the filter and the input, and then summing the results. The convolution operation can be expressed as follows:

(3) $$F\left(m,n\right)=\sum_{i}\sum_{j}I\left(m-i,n-j\right)\cdot K\left(i,j\right),$$

where *F* is the feature map output, *I* is the input, *K* is the filter/kernel, and (m,n) and (i,j) are indices for the spatial dimensions of the output feature map and the kernel, respectively. The convolution operation is applied with a stride of 1 and a padding of 1 to preserve the spatial dimensions of the input.

To reduce the spatial dimensions of the feature maps while preserving the most important information, the max-pooling operation [64] is performed on the output features.

The fully connected layers in VGG-19 are used to combine the features learned from the previous layers, by performing matrix multiplication between the input data and the weight matrix, followed by the addition of a bias term:

(4) Y=W·X+b,

where *Y* is the output, *W* is the weights matrix, *X* is the input, and *b* is the bias term.

Finally, the output of the last fully connected layer is passed through a softmax activation function to produce class probabilities.

The VGG-19 architecture is trained using a backpropagation algorithm to minimize a cross-entropy loss function:

(5) $$L\left(y,\hat{y}\right)=-\sum_{i=1}^{N}y_{i}\log\left({\hat{y}}_{i}\right),$$

where $L(y,\hat{y})$ represents the cross-entropy loss between the true and the predicted probability distribution and *N* is the total number of classes.

The weights and biases of the network are updated by the gradient descent optimization algorithm Adam [65], with lr=0.0001. By employing small filter sizes, deep architecture, and consistent design principles, VGG-19 has demonstrated exceptional performance in large-scale image recognition tasks.

#### 2.3.5. ResNet-101

ResNet-101 is a specific instantiation of Residual Networks (ResNets) [66], a family of deep CNNs. ResNet-101 consists of 101 layers, including convolutional layers, batch normalization layers, activation layers, and pooling layers, as well as residual connections.

A residual block presents the basis of ResNet architecture and can be represented as:

(6) y=F(x,W)+x,

where *y* is the output of the residual block, *x* is the input to the block, F(x,W) represents the residual operations applied, and *W* denotes the set of learnable weights associated with the block. The addition operation between F(x,W) and *x* forms a shortcut connection, allowing the gradients to flow more effectively during backpropagation, thus improving the optimization process.

ResNet-101 is structured as a series of stacked residual blocks, with each block containing multiple convolutional layers. The first few layers perform initial feature extraction, while subsequent layers capture increasingly complex patterns. ResNet-101 consists of four groups of residual blocks, with each stage featuring a different number of blocks and output feature map dimensions. Downsampling occurs between stages through pooling operations.

The model concludes with a global average pooling layer (GAP). For an input feature map *F* with dimensions H×W, the GAP operation can be defined as:

(7) $$GAP\left(F\right)=\frac{1}{H\times W}\sum_{i}^{H}\sum_{j}^{W}F\left(i,j\right),$$

where F(i,j) is the value at position (i,j) of the input feature map.

The GAP layer is followed by a fully connected layer that outputs class probabilities via a softmax activation function.

The goal of training ResNet-101 is to minimize a loss function, in this case a cross-entropy loss, using the Adam gradient descent optimizer with lr=0.0001. By employing residual learning and deep architecture, ResNet-101 has demonstrated exceptional performance on various computer vision tasks, including image classification and object detection, outperforming shallower CNN architectures and other classification methods not based on deep learning.

#### 2.3.6. DenseNet-169

DenseNet-169 is a deep CNN architecture that belongs to the Dense Convolutional Networks (DenseNets) family [67]. DenseNet-169 is particularly known for its efficient use of network parameters and its ability to alleviate the vanishing gradient problem, which enables the training of deep architectures while maintaining strong performance in various computer vision tasks, such as image classification and object detection.

The defining characteristic of DenseNets is the dense connectivity pattern, where each layer receives the feature maps from all preceding layers as input. This is in contrast to conventional CNNs, where each layer receives the inputs of its immediate predecessors. Mathematically, the output of the *l*-th layer in a DenseNet can be represented as follows:

(8) $$H_{l}=f_{l}\left(\left[H_{1},H_{2},\cdots ,H_{l-1}\right]\right),$$

where $H_{l}$ is the output feature map of the *l*-th layer, $f_{l}$ is the layer function (e.g., convolution, activation function), and $\left[H_{1},H_{2},\cdots ,H_{l-1}\right]$ denotes the concatenation of feature maps from layers 1 to (l−1).

DenseNet-169 consists of 169 layers and 4 dense blocks with varying numbers of densely connected layers and 3 transition layers interspersed between the dense blocks. The network starts with an initial convolutional layer, followed by dense blocks and transition layers in alternating order. The last dense block is followed by the GAP layer, which aggregates the feature maps into a compact representation, and a fully connected layer with a softmax activation function to generate class probabilities as the final output.

Similar to VGG-19 and ResNet-101, the DenseNet-169 architecture is also trained with the backpropagation algorithm to minimize the cross-entropy loss function, updating the weights and biases of the network by the gradient descent optimization algorithm Adam, with lr=0.0001. The dense connectivity pattern in DenseNet-169 facilitates efficient gradient flow and feature reuse, resulting in improved performance with fewer parameters compared to conventional deep CNN architectures.

## 3. Results and Discussion

In this section, we present the results in terms of accuracy percentages for different classification methods, different guidance types, different sampling frequencies, and different datasets. We also examine the statistical significance of the results obtained and then discuss the implications of the generalizations derived.

### 3.1. Comparison of Classification Methods and Preprocessing Frequency Bands with the ULM Dataset

The classification methods described in Section 2.3 are first compared on the ULM dataset with low-frequency features (0.2–5 Hz) and broad-frequency features (1–40 Hz). For the ULM dataset, as shown in Table 1, ResNet-101 achieved the highest accuracy (72.30%) in classifying EF vs. EE directions of MI. DenseNet-169 performed well with an accuracy of 66.24%, while all other methods were approximately at chance level (55%).

When comparing low-frequency and broad-frequency features, the highest accuracy was achieved with ResNet-101 with accuracy up to 69.82%. All methods performed similarly well with broad-frequency features compared to low-frequency features. Three simpler classifiers (sLDA, SVM, and RF) performed slightly better than with low-frequency features, and three CNN classifiers performed slightly worse, but all simpler classifiers performed about on par with a chance level on both frequency passbands.

In order to statistically analyze the effects of the method used and the frequency passband utilized in the preprocessing step, we performed a two-way repeated measures (RM) ANOVA based on the results from the ULM analysis. Namely, a 6×2 RM ANOVA was utilized, with Method (6 instances) and Passband (0.2–5 Hz, 1–40 Hz) being the within-subjects factors. The test yielded the following results:

- Mean classification accuracy differed statistically significantly between observed methods: F(5,70)=78.281, p<0.001. Post-hoc analysis with a Bonferroni adjustment confirmed that ResNet-101 statistically achieves the best results among all competitors (71.1%). The complete results of post hoc pairwise comparisons are given in Table 2.
- There is no significant effect of Passband on classification accuracy: F(1,14)=0.884, p=0.363>0.05. In other words, the difference in classification accuracy when preprocessing the ULM dataset using a low-frequency band (0.2–5 Hz) and using a broad-frequency band (1–40 Hz) is not statistically significant.
- The interaction between the factors Method∗Passband is not statistically significant: F(5,70)=1.816, p=0.121>0.05.

These results indicate that ResNet-101, which to our knowledge has not yet been used to classify MI EEG center-out movements of the same limb, shows promising results and can outperform other state-of-the-art classifiers. The superior performance of ResNet-101 can be attributed to several factors. First, ResNet-101 has a deep architecture with 101 layers, which allows it to capture complex patterns in the data. This is particularly important in the context of MI EEG classification, where patterns can be subtle and difficult to discern. The ability of ResNet-101 to capture such patterns is likely due to the presence of residual links that allow effective propagation of gradients during training. Second, ResNet-101 has a larger number of learnable parameters than the other classifiers tested. This enables it to learn more complex representations of the data and better adapt to the training set. Finally, ResNet-101 has been shown to perform well on a number of computer vision tasks, including image classification and object detection. This suggests that the architecture is well-suited to learning complex representations of visual data, which may be particularly relevant in the case of MI EEG classification, where data are represented as images. Compared with other relevant studies, Lee et al. [48] utilized channel-wise variational autoencoder-based CNN and achieved 60% accuracy in classifying the *elbow extension* against other same-limb MI tasks.

Our passband frequency analysis confirms previous findings [22,38] that the low-frequency delta band (0.2–5 Hz) fosters motor imagery information and that adding higher frequencies when dealing with same-limb different MIs does not improve classification accuracy. In fact, preprocessing data with broad-frequency features may actually decrease classification accuracy, as we pointed out in our results.

### 3.2. Comparison of Classification Methods, Guidance Types, and Preprocessing Frequency Bands with the KGU Dataset

To corroborate our results from the ULM dataset, we also tested our approach on the KGU dataset. Table 3 lists the descriptive statistics we obtained when testing the applied classification methods on the KGU dataset, examining the effects of guidance type and frequency passband simultaneously. As we can see, ResNet-101 achieved the highest accuracy for low-frequency features (0.2–5 Hz), up to 70.99%. DenseNet-169, sLDA, and SVM performed well with accuracies up to 65.61%, 64.07%, and 64.07%, respectively. RF was about at chance level, with accuracies up to 56.49%.

Comparing the guidance types in Table 3, we can see that the accuracy for the VtG condition is higher than for noVtG for all classifiers except VGG-19 and DenseNet-169. The highest accuracy for both VtG and noVtG was obtained with the ResNet-101 classifier (70.99% and 70.15%, respectively). sLDA and SVM showed the largest differences between the accuracies of the different guidance types, and both performed considerably better for VtG guidance. For broad-frequency features, all methods except ResNet-101 performed better for the VtG guidance type.

With respect to broad-frequency features, the highest accuracy was achieved with ResNet-101, up to 68.59%. All methods performed worse with broad-frequency features than with low-frequency features, with the exception of RF (which achieved near chance level accuracy for both types of features).

**Figure 4:**
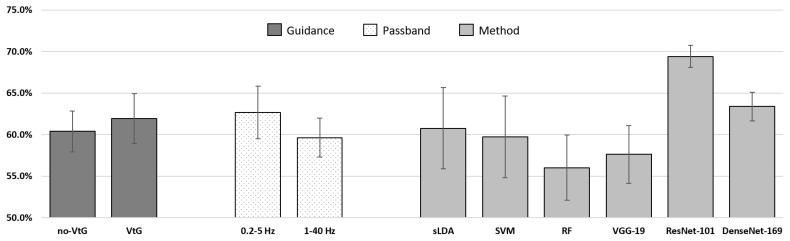
Descriptive statistics on classification accuracy for the KGU dataset.

In Figure 4, the data from Table 3 are presented in such a way that the values for classification accuracy are averaged according to all observed factors (type of guidance, passband frequency, classification method used). In order to statistically analyze classification accuracy results for the KGU data, we performed a three-way RM ANOVA. Namely, a 6×2×2 RM ANOVA was utilized, this time with Method (6 instances), Guidance (VtG, noVtG), and Passband (0.2–5 Hz, 1–40 Hz) being the within-subjects factors. The Greenhouse-Geisser ε correction for the violation of sphericity was applied when appropriate. In cases where a significant effect was found, post hoc pairwise comparisons with Bonferroni adjustment were utilized. The test yielded the following results:

- A significant effect of the Method on classification accuracy was again found: F(2.891,40.480)=37.574, ε=0.578, p<0.001. Similar to the case of the ULM dataset, ResNet-101 achieved the best classification accuracy (mean value of 69.4%). By far the worst accuracy, however, was obtained with the RF method (mean value of 56.0%). The results of the post hoc pairwise comparisons with Bonferroni adjustment are shown in Table 4.
- A significant effect of Guidance on classification accuracy was also found: F(1,14)=5.293, p=0.037<0.05. Hence, the classification accuracy when using vibrotactile guidance (VtG, 61.9%) is higher than in the case where there is no such type of assistance (noVtG, 60.4%). Although this difference may seem negligible in absolute terms, it is still statistically significant.
- The third observed factor, Passband, has a significant effect on classification accuracy as well: F(1,14)=18.3, p=0.001<0.05. If the data are preprocessed using a filter with a low-frequency band, a significantly higher accuracy is achieved (62.7%) than with a broad-frequency band (59.6%).
- None of the interactions between the observed factors are statistically significant:
  – Method∗Guidance: F(5,70)=1.410, p=0.231>0.05, ns.
  – Method∗Passband: F(5,70)=2.019, p=0.086>0.05, ns.
  – Guidance∗Passband: F(1,14)=0.043, p=0.839>0.05, ns.
  – Method∗Guidance∗Passband: F(2.540,35.553)=0.322, ε=0.508, p=0.777>0.05, ns.

The varying performance of our approach when using features of the ULM dataset compared to features of the KGU dataset may be due to several factors. First, the paradigms utilized in the two datasets are not identical. They differ in terms of timing (Figure 2 and Figure 3) and the specific movements imagined (e.g., *elbow flexion* and *elbow extension* for the ULM dataset vs. *up* and *right* for the KGU dataset). In addition, the paradigm of the KGU dataset included vibrotactile guidance on certain trials, which may have engaged participants more in the task. Another possible explanation could be the positive effect of visual guidance in the experiment of the KGU dataset compared to purely visual instructions in the experiment of the ULM dataset (as suggested by Yang et al. [68]). Furthermore, the availability of different electrode positions (as described in Section 2.1.2) could also contribute to the different performance results.

The best-performing applied method, ResNet-101, performs similarly well on both datasets, indicating the robustness of our preprocessing and classification pipeline. The proposed pipeline with ResNet-101 implementation was able to detect different MIs in the KGU dataset significantly better than the other compared methods and with comparable accuracy to the results obtained with the ULM dataset. These results also outperform other currently available CNN implementations in same-limb MI tasks [48] (60%, as discussed in Section 3.1) and other state-of-the-art commonly implemented classification techniques (60–64%) [37,38,39,41].

For the KGU dataset, statistical analysis of different passbands shows that using low-frequency features (0.2–5 Hz) in an experiment with vibrotactile guidance and with our preprocessing pipeline yields significantly better results than utilizing broad-frequency features (1–40 Hz). This is due to the fact that EEG amplitude modulations in the low-frequency range promote information about arm movement initiation and directional processing as an integral part of movement preparation [22].

The results of the statistical analysis of the different guidance types are consistent with our earlier findings [38], where it is shown that the condition VtG does not reduce the classifier’s ability to extract directional information. Rather, it has positive effects on the extraction of directional information with simple classifiers, such as sLDA and SVM. In this study, we have shown that the difference between guidance types is statistically significant when our preprocessing and classification are used.

Although the neurophysiological context of the different conditions was not the primary focus of this study, we were aware that movement-related cortical potentials associated with MI were present in both VtG and noVtG conditions [38]. However, in the VtG condition, the spatial prominence of these features was considerably higher. The more prominent features could provide clearer, more distinct signals to the classifiers, making them better in extracting directional information from the MI EEG center-out movements. This could make it easier for these simpler classifiers to identify the optimal separation, thus improving their performance. Complex classifiers, on the other hand, can model more intricate relationships and patterns within the data, even if the features are not as clearly separated. As a result, they may not benefit as much from vibrotactile guidance because they are already capable of handling less distinct features. However, they still benefit from the more distinct signals provided by vibrotactile guidance, so they can maintain their high performance regardless of the guidance condition (i.e., their performance does not decrease under VtG).

## 4. Conclusions

BCIs, based on MI, are a topic of interest to many researchers worldwide due to their wide range of practical applications. With advances in sensors, signal processing algorithms, and intelligent control solutions, the accuracy and reliability of BCI-based systems are improving every day. This paper provides a comprehensive overview of machine learning techniques that can be applied in the context of BCI MI and proposes a specific pipeline for data preprocessing and MI classification based on datasets derived from experiments with visual guidance, a combination of visual and vibrotactile guidance, or visual cues only. The preprocessed datasets were classified using sLDA, SVM, RF, ResNet-101, VGG-19, and DenseNet-169 methods.

Overall, our study compared several classification methods as well as different guidance types and sampling frequencies in classifying MI EEG center-out movements of the same limb. Our results showed that the proposed implementation of ResNet-101, after our preprocessing phase, achieved significantly better accuracy among the tested classifiers—up to 72.30% for low-frequency features of the ULM dataset and 70.99% for low-frequency features of the KGU dataset. These findings suggest that ResNet-101, which has not been previously used for this type of classification task, shows promising results and outperforms other state-of-the-art classifiers.

In addition, we confirmed the previous findings [22,38] that the low-frequency delta band (0.2–5 Hz) fosters motor imagery information and found that adding broad frequencies to the pipeline presented here does not improve classification accuracy but may actually decrease it.

From our statistical analysis, we also conclude that classification can be improved by using vibrotactile guidance (condition VtG). Thus, when vibrotactile guidance is used in a given experimental setup, one can expect significantly improved classification of directional information, with the positive effect being particularly pronounced for simple classifiers such as sLDA and SVM.

In conclusion, our study contributes to ongoing efforts to develop accurate and reliable brain–computer interfaces that could be used in motor rehabilitation and other applications. Our results suggest that ResNet-101 and low-frequency delta-band features should be considered in the development of BCIs for center-out MI of the same limb. Future research could investigate the use of ResNet-101 for other types of MI tasks and compare its performance with other state-of-the-art classifiers.

### Limitations and Future Work

Although vibrotactile guidance offers advantages for BCI-based systems, there are also potential limitations and obstacles to consider. One important factor is individual variability in response to vibrotactile stimuli. This can be influenced by variables such as age, sensory thresholds, and personal preferences. In addition, prolonged exposure to vibrotactile stimulation may lead to habituation, decreasing its effectiveness over time. Another potential obstacle is the technical difficulty of incorporating a vibrotactile feedback system into a portable, user-friendly device. The hardware must be durable and reliable and provide precise tactile stimulation. Adjusting the hardware to the operating position, while not very time-consuming, must also be considered a potential limitation.

It is important to note that both datasets were collected in offline settings rather than in online, real-time BCI experiments. Our current study targeted a single-session offline context and did not examine the possibility of intra-session performance degradation due to the nonstationarity of EEG signals, which is commonly observed in online studies where features may shift [29], and we recognize this as a potential limitation and important area for future research. Common Spatial Pattern and Filter Bank Common Spatial Pattern combined with Stationary Subspace Analysis are well-known techniques to address such performance issues (in addition to reducing the dimensionality of the data) [29], which typically results in higher classification accuracy. To improve the reliability and stability of our EEG-based BCI system over extended periods of use, the aforementioned techniques can be implemented and evaluated.

In the current offline analysis, we used an entire 2-s trial for CNN-based classification, which could introduce a delay of up to 2 s from the onset of MI in a real-time BCI scenario. This delay may potentially hinder user perception and control of the BCI system. As a result, we recognize the need to optimize the window size for online classification, balancing between the accuracy of classification and the speed of feedback to the user.

Finally, the ML pipeline presented in this paper has not been subjected to formal verification. Recently, formal methods have gained importance in the ML domain, where they are used to validate data preparation and training phases, as well as to verify machine learning systems [69]. These methods are based on mathematical models and logic and provide a way to verify the correctness of the system and evaluate its emergent behavior [70]. Because our research used existing datasets, well-known models and architectures, and widely available software libraries to implement these models, we did not consider formal methods to validate data preparation or model training. However, our solution could be strengthened by incorporating a formal approach to the verification of AI-based classification techniques.

The study limitations identified largely determine the plans for our future work. We will definitely focus on preparing and conducting an online BCI experiment to gain a more comprehensive insight into the effectiveness of the proposed solution. In addition, we plan to investigate how vibrotactile guidance affects the performance of our proposed pipeline, in a broader range of activities and with different MI tasks. This will allow us to understand the application-specific differences in the effects of various MI tasks and guidance types on classifier performance.

We also plan to focus on improving the classification accuracy of amplitude features (used in this work), power features (used in previous work [38]), and short-term entropy features. In particular, we want to use information entropy measures for different time-frequency distributions to investigate whether entropy change under different conditions (with or without vibrotactile stimuli) and different types of features (amplitude and power) can be utilized to improve classification methods and thus classification accuracy. Finally, we would like to examine the effects of increasing the size of our dataset through data augmentation.

## Figures and Tables

**Table 1:** Overall average (across all participants) classification accuracy of the different methods obtained on the ULM dataset with low-frequency (0.2–5 Hz) and broad-frequency (1–40 Hz) features. The best results for a given feature type are in bold.

	Accuracy (%)					
**Classification Method**	**sLDA**	**SVM**	**RF**	**VGG-19**	**ResNet-101**	**DenseNet-169**
EF vs. EE (0.2–5 Hz)	53.59	53.07	53.93	57.47	**72.30**	66.24
EF vs. EE (1–40 Hz)	54.75	55.47	54.03	56.24	**69.82**	62.94

**Table 2:** Pairwise comparisons of methods’ classification accuracy, as achieved on the ULM dataset. Values represent differences in accuracy means (%). Statistically significant differences (p<0.05) are emphasized accordingly *.

Method	sLDA	SVM	RF	VGG-19	ResNet-101	DenseNet-169
sLDA		−0.1	0.2	−2.7	−16.9 *	−10.4 *
SVM	0.1		0.3	− 2.6	−16.8 *	−10.3 *
RF	− 0.2	− 0.3		− 2.9	−17.1 *	−10.6 *
VGG-19	2.7	2.6	2.9		−14.2 *	−7.7 *
ResNet-101	16.9 *	16.8 *	17.1 *	14.2 *		6.5 *
DenseNet-169	10.4 *	10.3 *	10.6 *	7.7 *	−6.5 *	

**Table 3:** Overall average (across all participants) classification accuracy of the different methods obtained on the KGU dataset with low-frequency (0.2–5 Hz) and broad-frequency (1–40 Hz) features, shown by condition. The best results for a given feature type and condition are in bold.

Classification Method	Cond.	Accuracy (%)					
		sLDA	SVM	RF	VGG-19	ResNet-101	DenseNet-169
right vs. up (0.2–5 Hz)	VtG	64.07	64.07	56.49	59.29	**70.99**	65.31
	noVtG	60.44	59.64	55.87	60.05	**70.15**	65.60
right vs. up (1–40 Hz)	VtG	60.87	59.38	56.96	55.63	**67.93**	62.13
	noVtG	57.66	55.72	54.75	55.53	**68.59**	60.50

**Table 4:** Pairwise comparisons of methods’ classification accuracy, as achieved on the KGU dataset. Values represent differences in accuracy means (%). Statistically significant differences (p<0.05) are emphasized accordingly *.

Method	sLDA	SVM	RF	VGG-19	ResNet-101	DenseNet-169
sLDA		1.0	4.7 *	3.1	−8.6 *	−2.6
SVM	−1.0		3.7 *	2.1	−9.7 *	−3.6
RF	−4.7 *	−3.7 *		−1.6	−13.4 *	−7.4 *
VGG-19	−3.1	−2.1	1.6		−11.8 *	−5.8 *
ResNet-101	8.6 *	9.7 *	13.4 *	11.8 *		6.0 *
DenseNet-169	2.6	3.6	7.4 *	5.8 *	−6.0 *	

## Data Availability

The ULM dataset is from Ofner et al. [39] and is freely available in the BNCI Horizon 2020 http://bnci-horizon-2020.eu/database/data-sets (accessed on 28 April 2023). The KGU dataset is from Hehenberger et al. [38] and is available with permission from the authors.

## Abbreviations

The following abbreviations are used in this manuscript:

BCI	Brain–computer interface
CNN	Convolutional neural network
DNN	Deep neural network
DT	Decision tree
ECoG	Electrocorticography
EE	Elbow extension
EEG	Electroencephalography
EF	Elbow flexion
EMG	Electromiogram
ERD	Event-related desynchronization
ERS	Event-related synchronization
fMRI	Functional magnetic resonance imaging
GCN	Graph convolutional neural network
IC	Independent component
ICA	Independent component analysis
KGU	Kinesthetic guidance (dataset)
LDA	Linear discriminant analysis
LFP	Local field potential
MEG	Magnetoencephalography
ME	Movement execution
MI	Motor imagery
NF	Neurofeedback
noVtG	MI condition without vibrotactile stimulation
QP	Quadratic programming
ResNet	Residual Network
RF	Random forest
RM	Repeated measures
sLDA	LDA with shrinkage regularization
SMR	Sensorimotor rhythms
SSVEP	Steady-state visual evoked potentials
SVM	Support vector machine
ULM	Upper limb movement (dataset)
VtG	MI condition with vibrotactile stimulation
